# Method for the Isolation of “RNA-seq-Quality” RNA from Human Intervertebral Discs after Mortar and Pestle Homogenization

**DOI:** 10.3390/cells11223578

**Published:** 2022-11-11

**Authors:** Artemii A. Ivanov, Olga N. Leonova, Daniil S. Wiebe, Alexsandr V. Krutko, Mariya M. Gridina, Veniamin S. Fishman, Yurii S. Aulchenko, Yakov A. Tsepilov, Tatiana S. Golubeva

**Affiliations:** 1 Institute of Cytology and Genetics SB RAS, 630090 Novosibirsk, Russia; 2 Department of Natural Science, Novosibirsk State University, 630090 Novosibirsk, Russia

**Keywords:** cartilage RNA isolation, RNA-seq, RIN, intervertebral disc

## Abstract

The problem of isolating high-quality total RNA from intervertebral discs has no recognized solution yet. This is due to the extremely low content of live cells in the samples and the voluminous intercellular matrix. A variety of published protocols focused on isolating RNA from articular cartilage have recommended the use of expensive equipment, enzymatic matrix cleavage, or cell culture. In our study, we used a combination of the traditional QIAzol protocol (Qiagen, Germany) and RNEasy column purification (Qiagen, Germany) to obtain high-quality RNA from post-surgical intervertebral disc fragments. Only a mortar and a pestle were used for grinding, making our method particularly accessible. The isolated RNA with a RIN of ~7 is suitable for studying the expression profile of chondrocytes in situ. RNA-seq analysis of three samples demonstrated cell type ratios to be mostly relevant to intervertebral disc tissues, with over 70% of the chondrocytes of the three subtypes having an admixture of blood-related cells.

## 1. Introduction

RNA sequencing allows the phenotype to be determined at the level of individual cells and tissue fragments and is crucial for understanding the signaling pathways and mechanisms of disease development [1]. However, not all substrates are equally suitable for RNA isolation. Cartilage is regarded as unambiguously “problematic” due to the small number of cells and well developed intercellular matrix, which is hard to disintegrate [2,3,4]. Cells account for only 1–3% of the nucleus pulposus volume of the intervertebral discs, and with aging, the living chondrocytes are gradually replaced by dead ones [5], thereby limiting the amount of RNA that can be isolated.

Worldwide, about half of the adult population eventually experiences lower back pain caused by the degeneration of the intervertebral discs. This disease significantly reduces the quality of life of patients and is a major burden on the economy [6]. Studying the expression profile of chondrocyte RNA in the disease will help to better understand disc degeneration mechanisms at the cellular level and develop new treatment approaches, for example, using non-coding RNA [7,8].

Currently, active research on the intervertebral disc transcriptome is hampered by the difficulty of isolating RNA, not to mention other factors. All approaches that have been suggested to address this problem can be considered invasive, i.e., requiring enzymatic processing of living cells [3], which could potentially affect the expression profile. Even more “invasive” is culturing chondrocytes before RNA isolation [9]. Sequencing RNA isolated from instantaneously fixed cells in liquid nitrogen may help to validate or even refute the conclusions drawn in such works.

Studies on RNA isolation from knee cartilage are relatively numerous [2,4,10,11,12] and can be used as a guide when working with discs. However, articular cartilage is more structured and contains more chondrocyte-rich regions to be isolated and used, such as the superficial zone [13]. Unlike the studies on discs, the papers on articular cartilage describe the methods of RNA isolation without pretreatment, using a cryogenic mill [4], a technique that can solve the key problem of sample homogenization but requires specific equipment. 

Another variant of an expensive but effective solution of the RNA isolation problem is using the technologies of single-cell analysis, the potential of which has been shown by Zhang et al. [14]. Although requiring pretreatment and centrifugation of live cells, ScRNA-seq provides a detailed expression profile of different chondrocyte subpopulations.

Guided by already published protocols for RNA isolation from cartilage, we set out to develop an affordable protocol for isolating the total RNA from intervertebral disc fragments fixed in liquid nitrogen. This study provides recommendations for efficient grinding of frozen cartilage fragments up to 1.8 g using a mortar and pestle in liquid nitrogen. In addition, we present a modified protocol for the isolation of QIAzol with purification using the RNEasy Plant mini kit and test RNA sequencing results from several samples.

## 2. Materials and Methods

### 2.1. Ethics Statement

This study was approved by the independent ethics committee of the Novosibirsk Research Institute of Traumatology and Orthopedics on 2 October 2020. Informed written consent was obtained from each participant. All experiments and samplings were carried out in accordance with ethical and biosafety protocols approved by the committee.

### 2.2. Intervertebral Disc Sampling

Intervertebral disc fragment sampling was performed at the Novosibirsk Research Institute of Traumatology and Orthopedics according to a previously published protocol [15]. Disc fragments were obtained during surgical procedures for degenerative lumbar disease. All samples used in the study were collected over a time period 25 December 2020 to 16 April 2021. The time interval between disc fragment removal during surgery and freezing in liquid nitrogen was no more than 45 min. Further storage was performed at −80 °C. More details about the sampling can be found elsewhere [15]. 

### 2.3. Mortar and Pestle Homogenization

For grinding, we selected samples weighing at least 1 g, as the RNA in smaller samples had enough time to degrade due to the delay between resection and freezing. The sample was homogenized using a mortar and pestle, with an awl used for its fragmentation. Throughout the grinding process, the sample was coated with liquid nitrogen. With effective cartilage homogenization being a key step in the entire protocol, we chose to describe it in as much detail as possible. 

#### Grinding Technique

**Figure 1:**
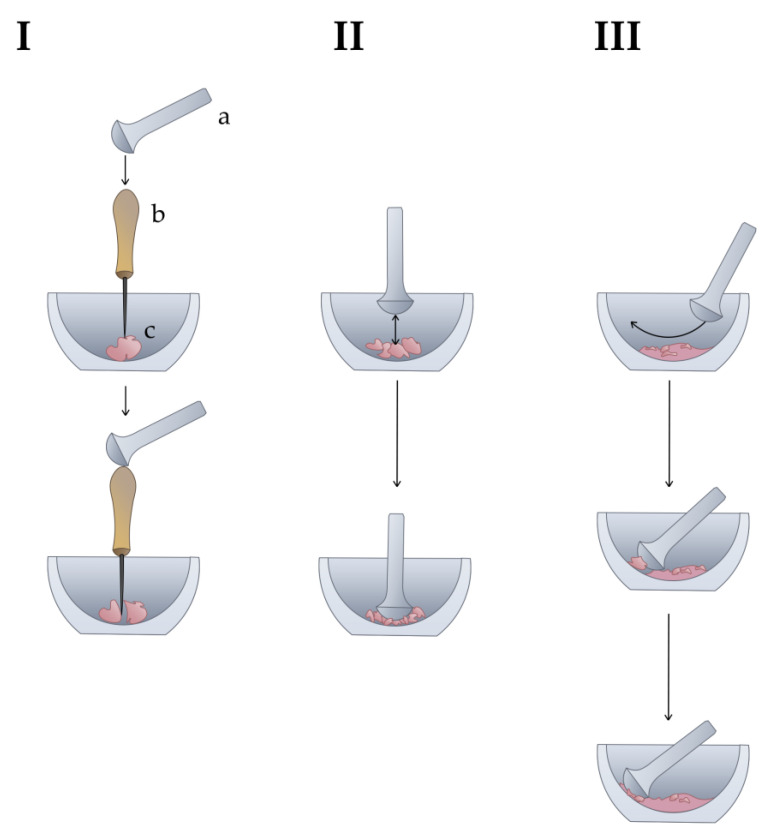
The technique of cartilage homogenization by mortar and pestle. The liquid nitrogen is not depicted to simplify the illustration. **I**: Fragmentation of a large piece of cartilage with an awl. a—pestle, b—awl, c—cartilage. **II**: Crushing of small fragments of cartilage. **III**: Crushing of the remaining small particles.

The homogenization process can be divided into three steps based on the crushing techniques involved (Figure 1).

First: using the awl as a chisel and the pestle as a hammer, one should crush the sample into small fragments (Figure 1I). 

Second: using the pestle strictly up and down, one should further crush the fragments. It is necessary to concentrate on one fragment at a time, making medium force beats with a low frequency, with the number of beats and their focus being more important than the pure force (Figure 1II).

Third: given that part of the sample has already been ground to a powdery consistency, for further efficient grinding it is necessary to run the pestle along the bottom of the mortar, taking care to trap larger granules and bring them to the side of the mortar (Figure 1III). Only when the granules are between the pestle and the side of the mortar can they be properly crushed by applying maximum force to the pestle while holding the mortar with the other hand. Homogenization can be stopped when the powder looks visually homogeneous.

In total, it takes approximately 25 min to homogenize one 1.8 g cartilage sample, assuming the experimenter has already mastered the technique. The ratio of time spent in each step was different depending on the texture and weight of the sample, on average corresponding to 1:2:3.

Upon achieving a powdery texture, the sample was grinded with QIAzol until homogeneous, then transferred to a 25 mL test tube, stirred to evenly coat all sides and lid, and left on ice until thawed.

### 2.4. RNA Isolation

QIAzol at a rate of 0.5 mL per 300 mg of the initial sample was added to a test tube with the sample thawed to viscosity. For better proteoglycan purification, the phase separation was carried out twice. After the first time separation, an equal volume of QIAzol was added to the aqueous phase, followed by incubation on ice for 30 min and re-centrifugation with chloroform. After the second time separation, the isolation was further carried out using the RNEasy spin column from the plant mini kit according to the manufacturer’s protocol. RNA elution was performed twice with 25 µL of RNase-purified water to increase the concentration. The aliquots for RIN determination and concentration measurements were selected immediately after isolation. RNA was stored at −80 °C. A complete protocol for RNA isolation is given in Table 1.

The concentration of isolated RNA was measured using a Qubit 4 Fluorimeter with an HS RNA Assay kit (Thermo Fisher Scientific, Waltham, MA, USA). A 2100 Bioanalyzer (Agilent Technologies, Santa Clara, CA, USA) and an RNA 6000 pico chip were used to assess the integrity and the length distribution of the isolated RNA. The analysis was carried out in the Genomics Center at the Institute of Chemical Biology and Fundamental Medicine, Siberian Branch of the Russian Academy of Sciences (Novosibirsk, Russia). Agilent software uses an algorithm to evaluate RNA integrity based on capillary electrophoresis data with a specific dye. As a result, the sample is assigned an RNA integrity number (RIN), ranging from 1 to 10, with 1 representing completely degraded RNA [16]. Only samples with a concentration greater than 10 ng/µL were selected for RIN determination.

### 2.5. Sequencing Libraries Preparation

Libraries were prepared with the MGIEasy RNA Directional Library Prep Set (MGI Tech, China) and sequenced using BGI Hong Kong facilities on the DNBseq platform in PE100 mode. Three samples total were sent to Hong Kong and arrived frozen on 3 December 2021. 

### 2.6. Quality Control of Sequencing Results

Quality control was performed using the FastQC tool [16]. Contamination analysis was performed using a fastq-screen tool [17] with the default database option. Raw reads were mapped onto the human genome (GRCh38) with the help of the STAR aligner [18]. TPM count normalization was performed using the TPMcalculator tool [19]. For deconvolution, the BisqueRNA R package was used [20], with scRNA-seq IVD atlas data as reference [21].

### 2.7. Statistical Analysis

MS Excel was used to calculate mean values, standard errors, and correlation coefficients. Exact formulas can be found in Table S1.

## 3. Results

### 3.1. Sampling and Homogenization

**Figure 2:**
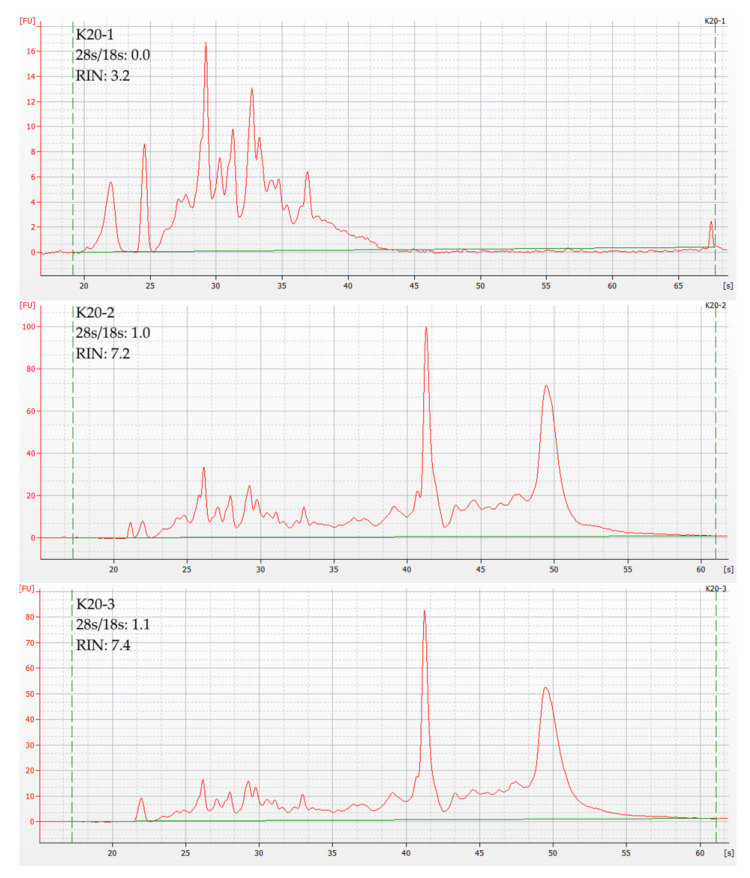
Graphical visualization of capillary electrophoresis results for derived cartilage samples K_20_12_25_03. Y-axis represents fluorescence intensity; X-axis represents time in seconds. Horizontal green line represents the fluorescence threshold; vertical dotted green lines mark the analyzed time period.

During the protocol’s development, it was found that only non-fragmented samples greater than 1 g in mass can consistently yield high-quality RNA. After homogenization, each such sample was divided into portions of ~300 mg mass, from which the RNA was extracted independently. This approach allows distinguishing between RNA degradation at the sampling/homogenization stage and directly at the isolation stage so that the protocol can be adjusted. In particular, we believe that it is at the RNA extraction stage that samples R_3_ and K_201_ (Table S1) were contaminated: for R_3_, the moment of contamination was logged during the run and was confirmed by the RIN difference (Table S1, File S1). Figure 2 shows the 2100 Bioanalyzer plots for K_201_, K_202_, and K_203_, from which the protocol error was deduced. Table 2 provides a complete list of the samples used.

RNA from samples 89_RAI, K_20_12_25_03, and N_20_12_25_01 was isolated in September, 2021. The remaining samples were taken for RNA isolation after we analyzed the sequencing results: in June and August 2022.

### 3.2. RNA Quality Control

With high accuracy of detection being a requirement, the HS RNA Assay kit for the Qubit fluorimeter was used for concentration measurements. When the protocol was tested on rough samples, the low RNA concentration was the major problem. Re-precipitation led to a loss of RNA quantity and quality. A threshold of 10 ng/µL was set for sequencing. To steadily overcome this threshold, the elution volume was reduced, and pair-wise pooling of the aqueous phases after the second separation and re-elution from the column were introduced. The average concentrations of the derivative samples are shown in Table 3. For a complete list of concentrations, see Table S1.

Excluding R_3_ and K_201_, the RIN of samples from the same cartilage varied only slightly, suggesting a predominant effect of sampling and homogenization procedures. For the original 2100 Bioanalyzer data, refer to File S1. The RIN of all derivative samples is given in Table S1. The average RIN for each sample is shown in Table 3.

The Pearson correlation coefficient between weight and mean C was r = 0.49 (Table 3), suggesting no significant correlation between these parameters. The correlation between mean RIN and weight (Table 3) can also be considered insignificant, r = 0.37. At the same time, the correlation coefficient between the average RIN and the average concentration of derivative samples was r = 0.77 (Table 3). In other words, when the sample is exposed to certain undesirable factors, both the amount and integrity of the extracted RNA are likely to decrease. We assume that the time between cartilage resection and placement in liquid nitrogen may significantly affect RIN and RNA amounts at the same time. However, measurements for individual samples were not feasible. Only the samples with a known RIN and concentration were used to calculate all correlations, i.e., 152_ZAI and 140_YAGi were excluded from the calculation. 

### 3.3. Quality Control of RNA-Sequencing Results

So far, we have obtained the sequencing results of derivative samples K20_3_, N_1_, and R_6_. One derivative sample per cartilage was selected for sequencing with the following parameters: RIN> 6, C > 10 ng/uL, total RNA > 200 ng.

**Figure 3:**
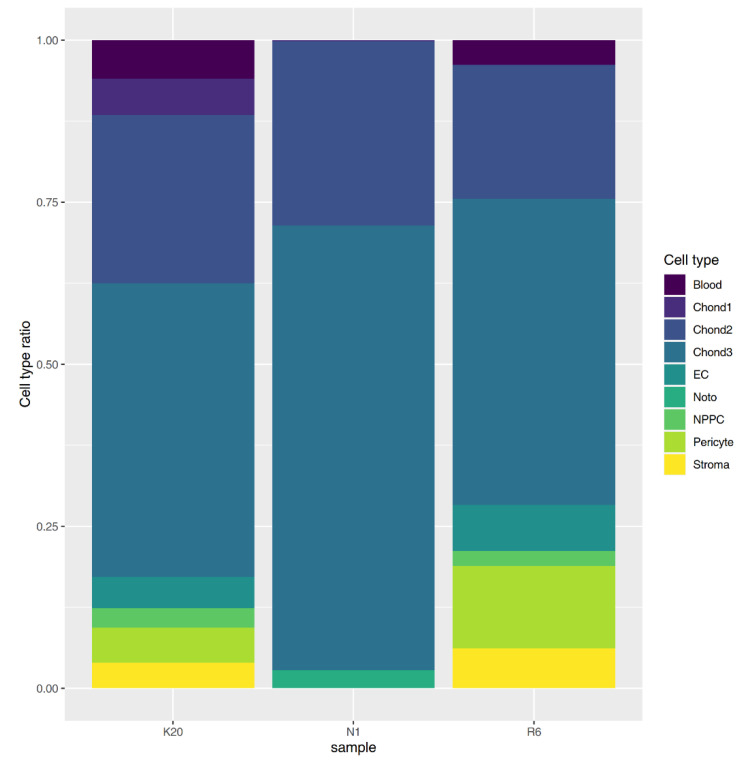
Intervertebral disc tissue transcriptome deconvolution by cell line.

Quality control with the FastQC tool demonstrated good overall quality, however, with a slight bias in the GC content for some samples. Neither contamination with rRNA nor with sequences derived from other species was observed. The cellular composition analysis in the experimental samples using the BisqueRNA software [20], with data from the single-cell transcriptomic atlas for intervertebral discs as a reference [21], demonstrated the ratios of cell types relevant to intervertebral disc tissue with a small admixture of blood cells (Figure 3), including over 70% of chondrocytes of the three types in each of the three samples. This indicates that the data obtained are suitable for intervertebral disc-specific expression assays. For detailed deconvolution data, see Table S2.

## 4. Discussion

The technique for total RNA isolation from intervertebral disc fragments presented in this paper is the only one that allows RNA-seq material to be obtained from cartilage using a mortar and pestle. Until now, it was thought that isolating high-quality RNA from cartilage required either pretreatment with enzymes [3,10], potentially altering the expression profile, or using specific equipment such as a cryogenic mill [4], which is not available to everyone due to its high cost. Moreover, a failure to isolate RNA after grinding with a mortar and pestle was reported in [3,4]. The reason for other researchers’ failures may have been due to an insufficient degree of grinding. For this reason, we have described the crushing technique in such detail as to allow one to achieve the result.

The use of the RNEasy spin column [4,11] after primary phase separation with TRIzol, as well as double phase separation [10], were previously described and successfully used, but they were not combined in a single protocol. We believe that given a high-quality homogenization, TRIzol and its analogs can provide protection against melting degradation and ensure effective lysis. At the same time, combining double phase separation with a column proves sufficient to isolate RNA for RNA-seq. 

The isolation protocol reported in the study [11] is very similar to the one we described. It involves using TRIzol for anti-degradation protection, purification on an RNEasy column, and an additional step of glycan purification. Zhang et al. used proteinase K for glycan purification, since their goal was to maximize the RNA yield. It is likely that treatment with proteinase K could replace the second phase separation in our scenario and lead to an increased yield, given that only about ½ of the aqueous phase volume is transferred to the column.

The work [11] is also of interest because the authors managed to obtain the same amount of RNA from 3 mg of cartilage as we have obtained from thousands of times larger samples. Due to the fact that they worked with animals, they succeeded in providing ideal conditions for sampling and immediate excision of the fragment of interest. In our case, it is not so much the very low number of chondrocytes in the tissue that dictates the need to use cartilage >1g, but rather the risk of RNA degradation in smaller samples due to suboptimal extraction. Were it possible to reduce the delay between surgery and freezing, as in [4], the sample size for RNA isolation according to our protocol could probably be reduced.

Alternatively, separating one large sample into several isolates, as we have done, proves to be effective even if the cartilage has been kept at room temperature for up to 45 min. Despite the increased costs of reagents and columns, our solution is noise-tolerant: it allows one to select a sample that is suitable for sequencing in almost all cases. Also, one can distinguish whether the low RIN is due to contamination during isolation or to the sub-optimal collection and poor homogenization of the material. In the first case, the low quality will only be characteristic of individual samples (Table S1).

Heather et al. [4] point to a critical step in freezing in liquid nitrogen: the exposure time of cartilage at room temperature should not exceed 3 min. Our results show that this statement is primarily true for small or fragmented samples that were not suitable for RNA isolation in our case [data not shown]. The main advantage of this work over other methods for isolating RNA from cartilage is its availability. When compared to cryogenic mills and single-cell technologies, TRIzol (or its equivalent) and isolation columns are available to almost any laboratory engaged in RNA isolation. Moreover, our method limits the effect of external factors on chondrocytes only to the rate of post-operative freezing. To conclude, our method of RNA isolation from chondrocytes can enhance the potential for the accumulation of accurate transcriptomic data on the causes and pathogenesis of intervertebral disc degeneration worldwide.

## Figures and Tables

**Table 1:** The full protocol for RNA isolation.

Methodology for Lumbar Disc RNA Isolation after Mortar and Pestle Homogenization
1. Grind the sample using a mortar and pestle (Figure 1).
2. Add QIAzol* to the mortar at a rate of 0.5 mL per 300 mg of frozen sample and grind to homogeneity in liquid nitrogen.
3. Pour into a 25 mL Eppendorf test tube, allow the nitrogen to evaporate, then close and mix by turning the tube. The powder should evenly coat the sides of it.
4. Allow it to remain on the surface of the ice until thawed. Vortex once every 5 min.
5. After thawing to viscosity, add another 0.5 mL of QIAzol for every 300 mg of the frozen sample. Vortex for 30 s, then incubate on ice for 5 min.
6. Remove the tip of the automatic pipette spout with sterile scissors. Pour 1 mL lysate into 1.5 mL microcentrifuge tubes. Add 0.2 mL of chloroform to each tube.
7. Stir by turning the tubes for 30 s, then vortex for 1–2 s. Incubate for 2 min on ice.
8. Centrifuge for 20 min at 15,000× *g* and 4 °C. At this stage, the white suspension in the water phase is acceptable if one can see through it. If not, repeat the centrifugation.
9. Carefully transfer the aqueous phase (usually ~400 µL) to new 1.5 mL microcentrifuge tubes and add an equal volume of QIAzol. Stir by inverting the tubes.
10. Incubate for 30 min on ice in the dark.
11. Add 0.2 volume of chloroform (usually ~160 µL). Repeat step 7.
12. Centrifuge for 20 min, 15,000× *g*, 4 °C. Repeat if necessary until a white suspension precipitates.
13. Transfer half of the aqueous phase from each tube (usually ~250 µL) to new 1.5 mL microcentrifuge tubes. To reduce column consumption and increase RNA concentration, it is recommended at this stage to combine the aqueous phase from every two tubes (4 tubes before phase separation–> 2 tubes after, and two RNEasy spin columns respectively).
Then the RNEasy plant mini kit (Qiagen) is used.
14. Add 0.5 V of ethanol to each tube and mix by pipetting. Transfer to the RNEasy spin column (pink).
15. Centrifuge for 15 s at 10,000× *g* and 15 °C. Discard the flow-through.
16. Add 700 µL RW1 buffer. Repeat step 15.
17. Add 500 µL RPE buffer (check that ethanol was added). Repeat step 15. Repeat the washing step with the RPE buffer twice.
18. Transfer each column to a new collection tube. Centrifuge for 1 min at 12,000× *g* and 15 °C.
19. Place the columns in new 1.5 mL microcentrifuge tubes and gently apply 25 µL of RNAse-free water to the membrane. Incubate for 1 min at room temperature.
20. Centrifuge for 30 s at 10,000× *g* and 15 °C.
21. Re-apply the eluate to the membrane to increase the yield. Incubate for one minute. Repeat step 20. Collect aliquots for concentration and RIN measurements.
22. Store the isolated RNA at −80 °C.

* QIAzol (Qiagen) is specified in the protocol, as we used it in our study. However, the more common TRIzol (Thermo Fisher Scientific) is also suitable.

**Table 2:** List of samples.

Sample Code	Sample Weight (g)	Derivative Samples *
K_20_12_25_03 **	1.4	K20_1_ – K20_3_
N_20_12_25_01 **	0.7	N_1_, N_2_
89_RAI **	1.8	R_1_ – R_6_
124_MAA	1.8	MAA_1_-MAA_3_
143_KEV	1.2	KEV_1_, KEV_2_
134_TSA	1.2	TSA_1_, TSA_2_
137_SGN	1.2	SGH_1_, SGH_2_
131_MTU	1.9	MTU_1_-MTU_3_
152_ZAI ***	1.8	ZAI_1_-ZAI_3_
140_YAGi	2.1	YAG_1_-YAG_3_

* The number of derivative samples is equal to 0.5 V QIAzol per mL used in steps 2 and 5 of Table 1, except for those marked with “**”. ** Earlier samples that were sent for RNA-seq. The aqueous phases were not combined in pairs after the second phase separation. The number of derivative samples is equal to the amount of QIAzol in mL as used in steps 2 and 5 of Table 1. No restriction on mass or sample integrity was applied. *** Fragmented and thus failed to pass the concentration threshold, so no RIN data are available.

**Table 3:** Mean concentrations and RINs.

Sample Code	Sample Weight (g)	Mean C ± SEM (ng/uL)	Mean RIN ± SEM
K_20_12_25_03	1.4	71.2 **±** 17.2 *	7.3 ± 0.1 *
N_20_12_25_01	0.7	8.5 **±** 1.7	6.7 ± 0.2
89_RAI	1.8	49.4 ± 3.4 *	6.9 ± 0.1 *
124_MAA	1.8	69.1 ± 4.0	8.5 ± 0.3
143_KEV	1.2	14.8 ± 3.6	7.0 ± 0.05
134_TSA	1.2	27.3 ± 2.3	6.7 ± 0.1
137_SGN	1.2	18.5 ± 0.4	6.3 ± 0.2
131_MTU	1.9	12.8 ± 2.5	6.5 ± 0.2
152_ZAI	1.8	6.1 ± 1.4	N/D **
140_YAGi	2.1	29.4 ± 7.1	7.8 ***
All	-	32.4 ± 4.7 *	7.1 ± 0.2 *

* K20_1_ and R_3_ were excluded due to contamination; ** Concentration threshold was not passed; *** RIN was measured for only 1 sample out of 3

## Data Availability

Not applicable.

## Supplementary Materials

The following supporting information can be downloaded at: https://www.mdpi.com/article/10.3390/cells11223578/s1, Table S1: Derivative sample RINs and concentrations; Table S2: Deconvolution data; File S1: Agilent bioanalyzer data archive.
